# 580 Regional Anesthesia for Autograft Donor Sites

**DOI:** 10.1093/jbcr/irae036.214

**Published:** 2024-04-17

**Authors:** Kristina Canady, Jacob M Loyd, Scott W Mueller, Cameron Gibson, Brianna Moorehead, Arek J Wiktor

**Affiliations:** University of Colorado Health, Denver, CO; University of Colorado School of Medicine, Denver, CO; UCHealth and University of Colorado School of Pharmacy, Aurora, CO; University of Colorado, Aurora, CO; Uchealth Burn Center, Aurora, CO; University of Colorado Hospital, Aurora, CO; University of Colorado Health, Denver, CO; University of Colorado School of Medicine, Denver, CO; UCHealth and University of Colorado School of Pharmacy, Aurora, CO; University of Colorado, Aurora, CO; Uchealth Burn Center, Aurora, CO; University of Colorado Hospital, Aurora, CO; University of Colorado Health, Denver, CO; University of Colorado School of Medicine, Denver, CO; UCHealth and University of Colorado School of Pharmacy, Aurora, CO; University of Colorado, Aurora, CO; Uchealth Burn Center, Aurora, CO; University of Colorado Hospital, Aurora, CO; University of Colorado Health, Denver, CO; University of Colorado School of Medicine, Denver, CO; UCHealth and University of Colorado School of Pharmacy, Aurora, CO; University of Colorado, Aurora, CO; Uchealth Burn Center, Aurora, CO; University of Colorado Hospital, Aurora, CO; University of Colorado Health, Denver, CO; University of Colorado School of Medicine, Denver, CO; UCHealth and University of Colorado School of Pharmacy, Aurora, CO; University of Colorado, Aurora, CO; Uchealth Burn Center, Aurora, CO; University of Colorado Hospital, Aurora, CO; University of Colorado Health, Denver, CO; University of Colorado School of Medicine, Denver, CO; UCHealth and University of Colorado School of Pharmacy, Aurora, CO; University of Colorado, Aurora, CO; Uchealth Burn Center, Aurora, CO; University of Colorado Hospital, Aurora, CO

## Abstract

**Introduction:**

Pain control remains a significant problem in burn patients. To some extent, increased pain can be predicted during the first 24-48 hours following donor site placement and during donor site dressing takedowns. Our objective was to evaluate the utilization of regional anesthesia in the form of single shot nerve blocks (SSNB) on pain in the perioperative setting of a donor site placement and prior to a donor site takedown.

**Methods:**

We performed a retrospective cohort study with prospective secondary survey examining SSNB effectiveness at our ABA verified burn center. Patients were candidates for SSNB if autograft donor sites were predicted to remain within femoral nerve sensory distribution zones. Opiate requirements 48 hours prior to and following SSNB as well as procedural requirements during the previous major wound care session or surgical case were collected. Procedural and overall opiate requirements were compared with and without a SSNB. Similar data were collected in a cohort of patients who did not receive a SSNB but otherwise would have qualified for one. A matched-pairs analysis compared each patient against themselves (pre-post). Surveys were administered to patients and staff on perceived effectiveness and pain scores.

**Results:**

A total of 13 blocks were administered, 9 for a donor site take down. Patients were predominantly male (n=8) with a median (interquartile range) age of 44 (28.5, 58.5) and TBSA 3% (1.9, 6.8). SSNB was associated with less intra-patient procedural fentanyl and a trend in less midazolam requirements between matched groups, mean difference of 89.4mcg (p=0.049) and 0.62mg (p=0.055), respectively. Eleven patient and six staff surveys were completed. There were no significant differences in patient reported pain/effectiveness between matched groups (p=0.13). 4 patients reported zero pain, 2 had 50% or greater decrease. However, 4 patients reported increased pain/discomfort: 1 due to SSNB administration and 3 due to “heavy or numb sensation”, but not pain itself. Staff perception of pain control improved from agreed (83.3%) to strongly agreed (66.7%), p=0.04. Overall total opiate exposures were not different pre-post block at 0-24 or 24-48 hours, p=0.2 and p=0.14, respectively.

**Conclusions:**

Utilization of regional anesthesia in a burn specific population may reduce the need for procedural opiates in anticipation of painful procedures. However, patient perceptions of pain/discomfort/numbness are varied, and education regarding expectations is warranted. Further research is needed to fully assess the effectiveness of SSNB for burn patients.

**Applicability of Research to Practice:**

In selected patients, SSNB can be a useful adjunct to reduce procedural opiates.

